# The insecure airway: a comparison of knots and commercial devices for securing endotracheal tubes

**DOI:** 10.1186/1471-227X-6-7

**Published:** 2006-05-24

**Authors:** Paris B Lovett, Alexander Flaxman, Kai M Stürmann, Polly Bijur

**Affiliations:** 1 Department of Emergency Medicine, NewYork Presbyterian Hospital, NY, USA; 2 Department of Emergency Medicine, Beth Israel Medical Center, NY, USA; 3 Albert Einstein College of Medicine, NY, USA

## Abstract

**Background:**

Endotracheal Tubes (ETTs) are commonly secured using adhesive tape, cloth tape, or commercial devices. The objectives of the study were (1) To compare degrees of movement of ETTs secured with 6 different commercial devices and (2) To compare movement of ETTs secured with cloth tape tied with 3 different knots (hitches).

**Methods:**

A 17 cm diameter PVC tube with 14 mm "mouth" hole in the side served as a mannequin. ETTs were subjected to repeated jerks, using a cable and pulley system. Measurements: (1) Total movement of ETTs relative to "mouth" (measure used for devices) (2) Slippage of ETT through securing knot (measure used for knots).

**Results:**

Among commercial devices, the Dale^® ^showed less movement than other devices, although some differences between devices did not reach significance. Among knots, Magnus and Clove Hitches produced less slippage than the Cow Hitch, but these differences did not reach statistical significance.

**Conclusion:**

Among devices tested, the Dale^® ^was most secure. Within the scope offered by the small sample sizes, there were no statistically significant differences between the knots in this study.

## Background

Unplanned extubation (UE) is a life-threatening event, and in recent years has been a focus of continuous quality improvement (CQI) programs [1-7]. While CQI programs and research have improved the care of the intubated patient, relatively little attention has been given to experimental comparisons between methods for endotracheal tube (ETT) securement.

UE is a multi-factorial problem, and one that affects many disciplines, notably anesthesia, critical care, and pre-hospital and emergency medicine. While intubated patients may spend less time in Emergency Departments (EDs) than they do in Intensive Care Units (ICUs) or Operating Rooms (ORs), the ED stay represents a vulnerable period, and UE is a topic worthy of the attention of Emergency Physicians (EPs).

Our study makes two sets of comparisons, both looking at rates of failure for different methods of ETT securement. The first comparison is between six commercial devices manufactured specifically for securing ETTs. The second comparison looks at three different knots used to tie ETTs in place, using non-adhesive cloth tape (also known as twill tape, or umbilical tape).

Our hypotheses were (1) that commercial devices would differ in the degree of total ETT movement occurring, and (2) that knots would differ in the degree of slippage occurring.

## Methods

### Study design and protocol

Our study followed an in-vitro experimental design. Following the lead of Patel et al. [8] we used a 17 cm diameter PVC tube as a mannequin, to simulate the adult intubated patient. A 14 mm diameter hole was cut in the side to simulate the mouth. ETTs were inserted to a depth of 22–28 cm and secured in place using the various methods under study. We used 7.5 mm internal diameter ETTs.

The authors invited many manufacturers of commercial ETT stabilization devices to supply units for testing, and received samples of six devices. Manufacturers who did not supply samples before deadline were not included in the study. The devices supplied were: Comfit™ (Ackrad), Stabiltube™ (B&B Medical), Tube Restraint^® ^(ErgoMed), ETAD™ (Hollister), Thomas ST™ (STI Medical) and Dale^® ^ETT Holder. Devices were supplied in varying quantities, and these quantities determined sample sizes.

All commercial devices were applied after carefully consulting the accompanying manufacturers' instructions, both printed and audiovisual. Some devices were accompanied by instructions for preparing the ETT with a chemical swab, and these instructions were followed.

For the knot comparisons our study used 12 mm width synthetic twill as the cloth tape. Knots were tied firmly enough to make an impression upon the ETT. The two free ends of the cloth tape were tied together at the side of the PVC tube. The knots we tested were the Clove Hitch (Clove), Magnus Hitch (Magnus), and the Cow Hitch (Cow). The Cow Hitch is also known as the Lark's Head Hitch.

All devices and all knots were wet with saline after being secured in place. This was done in order to simulate the moist environment in which most ETTs secured in the ED must function.

A single operator was responsible for securing all knots and devices, in order to standardize trial conditions.

The external end of the ETTs was then attached to a cable and pulley system, with a spring in series (spring constant 150 N/m). The other end of the cable was attached to a weight. The weight could be gently lowered until the cable and spring were at full length without experiencing any drop thus producing a static load upon the ETT and its securement. The cable was then pulled back, and the weight dropped a distance of 12 inches until the cable snapped taught and jerked on the ETT. This produced a dynamic load, or jerk.

Our study involved no human subjects and was exempt from IRB approval. All testing was performed in December, 2002.

### Measurements

We tested devices by applying a series of loads to the endotracheal tube, then measuring any movement which occurred following the load. Loads were applied using a range of weights (see below). First a static load was applied (weight attached, without any jerk). Then a series of dynamic loads was applied (12 inch drop). After applying each load, the position of the tube and the knot/device were checked. The position of the knot or device on the tube was noted. Also the position of the tube relative to the "mouth" was noted.

Based upon these measurements we derived the following measures (1) Total movement of the tube (movement of tube relative to mouth) and (2) Slippage (movement of knot or device along the tube; accounting for a portion of total movement).

We first performed a series of pilot tests, to determine which loads produced maximal differentiation in performance between devices and knots. We pilot tested weights ranging from 1 lb to 25 lbs. We ultimately settled upon using loads of 2.5 lbs and 5 lbs to test the commercial devices, and 10 lbs to test the twill tape with knots.

In the study proper, we subjected all devices and knots to one static load followed by 15 dynamic loads. As the study end point for devices, we compared total movement. As the study endpoint among knots we compared slippage (movement of the knot along the tube). This is a better measure of knot performance than total movement, as it distinguishes movement due to material stretch (not related to the knot) from movement due to slippage of the knot upon the tube (the main point of interest). We also measured total stretch, in order to confirm that the stretch component of movement was the same whichever knot was tied.

All measurements were taken after 6 jerks and after 15 jerks.

### Statistical analysis

Measurements of total movement for commercial devices were compared using the Kruskal Wallis test. Individual pairs of devices were then compared using the Mann-Whitney U test, with Bonferroni correction for multiple comparisons.

The same procedure was followed in comparing slippage among knots, and in comparing total movement among knots.

Given the small numbers, these non-parametric tests were considered most appropriate.

No power analysis was performed, because sample sizes were determined by supplies of devices made available by manufacturers.

## Results

### Commercial devices

The Dale^® ^device showed less total movement than the other devices, at both 2.5 lbs and 5 lbs, when measured after 6 jerks and after 15 jerks. Kruskal Wallis tests showed that, overall, the devices performed differently. We then compared the device with the lowest median movement (Dale) against each of the other devices using Mann-Whitney U tests. Some of differences in movement between the Dale and the other devices were statistically significant, others were not.

Table 1 shows the median (minimum, maximum) total movements for each device at 2.5 lbs (top), and at 5 lbs (bottom), and shows P-values for the differences between the Dale and the other devices.

**Table 1:** 

Device	N	Median Total Movement (Min, Max) at 6 jerks	P-Value for Comparison with Dale at 6 jerks	Median Total Movement (Min, Max) at 15 jerks	P-Value for Comparison with Dale at 15 jerks
**(A)**					

Dale	5	0.25 (0.00, 1.00)	-	0.50 (0.00, 1.25)	-
Comfit	4	1.75 (1.50, 13.25)	0.016*	2.00 (1.50, 26.00)	0.016*
Stabiltube	5	3.00 (2.25, 3.50)	0.008	3.75 (2.50, 6.00)	0.008
ETAD	6	25.75 (23.25, 26.75)	0.004	25.75 (23.25, 26.75)	0.004
Tube Restraint	5	5.00 (0.00, 6.50)	0.095*	9.50 (7.00, 19.50)	0.008
ThomasST	8	2.88 (1.50, 25.50)	0.002	7.63 (3.75, 27.00)	0.002

**(B)**					

Dale	5	2.00 (1.75, 2.75)	-	3.00 (2.50, 3.50)	-
Comfit	5	16.00 (6.00, 26.50)	0.008	26.25 (8.50, 26.50)	0.008
Stabiltube	5	25.50 (25.25, 26.75)	0.008	25.50 (25.25, 26.75)	0.008
Tube Restraint	3	23.25 (7.25, 25.50)	0.036*	23.25 (10.25, 25.50)	0.036*

Part (A)

Testing of commercial devices at 2.5 lbs.

Differences among all devices significant (p < 0.01 for both 6 jerks and 15 jerks)

P-Value ≤ 0.010 considered significant for paired comparisons. * = not significant

Part (B)

Testing of commercial devices at 5 lbs.

Differences among all devices significant (p = 0.01 for both 6 jerks and 15 jerks)

P-Value ≤ 0.017 considered significant for paired comparisons. * = not significant

### Knots

The Clove Hitch and Magnus Hitch showed less slippage than the Cow Hitch in the samples; however these differences were not significant. Results for the knots are shown in Table 2. There were also no significant differences in total movement between knots.

**Table 2:** 

Knot	N	Median Total Movement (Min, Max) at 6 jerks	Median Total Movement (Min, Max) at 15 jerks
Clove	4	2.00 (0.50, 5.25)	2.38 (0.75, 24.75)
Magnus	5	2.75 (1.75, 4.00)	4.00 (2.25, 5.75)
Cow	11	5.50 (0.50, 25.00)	6.00 (2.25, 25.00)

Testing of knots in cloth tape at 10 lbs.

Differences among all knots not significant (p = 0.13 for 6 jerks and p = 0.055 for 15 jerks)

P-Values for all paired comparisons of knots > 0.017 (not significant)

## Discussion

UE is only one of a long list of complications of endotracheal intubation. Even assuming proper tube placement and cuff pressure, there are many potential morbidities: tissue trauma may affect the ears and scalp (from ties passing over them); nasal tissues; lips, oral mucosa, gingivae, pharynx, glottis and subglottic tissues. Leverage upon the external end of the ETT may traumatize the lower respiratory tract. ETT securement methods may produce difficulties in access for suction of the upper and lower respiratory tract. Infections of the sinuses as well as other areas of the respiratory tract are common. There is also the potential for further traumatizing burns and fractures [9-15].

Among all these potential problems, UE is one which Emergency Physicians and Pre-Hospital providers must grapple with on unique terms. In Pre-Hospital and ED conditions, patients may not always be adequately sedated, they may be moved frequently, and physical monitoring may not always be consistent. Tube dislodgement during Pre-Hospital transport has been noted as an important problem [16].

The incidence of UE varies widely between institutions, and over time, and has been reduced in some instances by CQI programs 1. Some reports have given rates as low as 0.3% in the wake of CQI efforts. Estimates in the Intensive Care Unit (ICU) and anesthesia environments during the 1980s and 1990s have offered wide ranges for UE, from 1.6% to 21% of all intubated patients [2-7,12,15]. The same reports discuss risk factors for UE, some well-established and others less so. These include inadequate sedation, inadequate restraint, agitation or confusion; delay of extubation; careless movement of the patient's head for suctioning, or during procedures and imaging studies, and careless transport procedures; improper cuff inflation, improper placement of the ETT, failure to shorten the portion of the ETT outside of the patient, and failure to stabilize the tubes and machinery attached to the ETT. Factors over which there is disagreement include time of day or shift; how busy the staff are; older patients; and prolonged intubations. There is general agreement that the method of tube securement is important.

Given the importance of adequate sedation and restraint, these reports should be carefully considered by emergency physicians. After a rapid sequence induction, there is the potential for omission or delay in giving ongoing sedation, analgesia and, when appropriate, paralysis.

UE is a potential disaster, and loss of airway is not the only complication of UE. Other morbidities include tissue trauma to the face and airway; bronchospasm; aspiration pneumonia; and dysrhythmias [3,5,12,15]. Mort reports that less than one third of re-intubations following UE are problem-free [2]. Serious morbidities such as brain injury and death may impact less than 5% of UE situations, but other morbidities may be under-reported.

Not every patient who suffers UE will require re-intubation. Reported rates of re-intubation cover an extremely wide range, from 15% to 78%, again in the anesthesia and ICU context. There are definable risk-factors for the need to re-intubate, the most well-established of which is accidental extubation by staff (during suctioning, transport, procedures, etc.) as opposed to self-extubation. Self-extubation is much more common than accidental extubation (approximately 75–85% of all UE) [1,2,4-6]. Other factors predicting the need for re-intubation include high FiO2 requirements; patients who have been ventilated for longer periods; patients who were not being weaned at the time of UE; and patients with depressed levels of consciousness or heavy sedation.

Emergency physicians can afford a more focused approach to preventing UE than other disciplines, because there is a narrower range of factors in our immediate control. Sedation, analgesia, paralysis and restraint must be given careful attention. Support for machinery attached to the ETT is important. Care during suctioning, procedures and transport must be particularly emphasized in the bustle of ED work. This paper focuses on the final factor: ETT securement.

Methods for securing ETTs can be broadly classified into four groups: First, there is adhesive tape, applied to the face and head in a variety of ways; Second is cloth tape, tied around the tube and around the neck and occiput. Third, there are specialized devices, both purpose-built commercial devices, and devices or arrangements fashioned by clinicians using a variety of hospital products. Finally, there are securement methods usually reserved for specialized situations such as facial burns, fractures and oral and maxillofacial surgery.

The first two methods, cloth tape and adhesive tape, are by far the most commonly employed. Earlier studies have compared these two methods and have found them to be equally secure [15,17]. There is also a study comparing the security of adhesion between different brands of adhesive tape and different ETT brands [18]. We chose not to include adhesive tape in this study both because of the existing evidence, and because it would have been hard to create a valid experimental model using a mannequin. The surface characteristics of the mannequin do not offer a valid model for adhesion to skin.

One thing that stands out both on reviewing the literature and in clinical practice is the highly detailed and personal contrivances fashioned by individual clinicians for securing ETTs, both with adhesive tape and with assemblages of equipment. Airway security seems to bring out the inventor, the home handyman, and the obsessive-compulsive in physicians and nurses. Taping rituals more elaborate than origami turn up in a literature search [8,19-22]. One of the most well accepted and enduring is that described by Gregory [23] for securing ETTs in neonates. There are also endless reports of assemblages of wire, felt, Elastoplast™, suture material, metal nuts, Velcro™, plastic tubing, molded rubber, tongue depressors, umbilical clamps, face masks, safety pins, o-rings and cable-ties [9,11,24-32]. Many of these reports reflect a painstaking and idiosyncratic enthusiasm.

In the context of facial burns and injuries, specialized means for securing ETTs are appropriate. Many of these approaches are described in the literature of oral and maxillofacial surgery, otolaryngology, and pediatrics, but they are potentially useful for emergency physicians with high trauma caseloads. The approaches include intra-oral wire or suture, affixing the ETT to teeth [8,33,34]; arches mounted over the face [11,25]; and circummandibular sutures [35,36].

There are reports comparing commercial devices with adhesive tape, and comparing different commercial devices with each other, but these are few.

Commercial devices are used in only a small percentage of intubated patients. According to unpublished commercial estimates provided by manufacturers (2003), somewhere between 13 and 20 million intubations are performed annually in the United States. Emergency and intensive care departments purchase approximately 3 million ETTs. The total market for commercial ETT securement devices is less than 500,000 units. This would imply that commercial devices are used in less than 5% of intubations.

In our study, the lack of variation in the results for the Dale^® ^is noteworthy. We would suggest that commercial devices are under-utilized in the ED, given the effectiveness of the Dale^® ^demonstrated in this study.

Cloth tape is used in close to half of all intubated patients, and yet little investigation has occurred on the question of how to tie it. Indeed, a prominent authority on knotting made the following comment: ". . . evidence is emerging that surgeons are not selective knot tyers. Practiced? Yes. Dextrous? Yes, very. Choosy and knowledgeable? Perhaps not. If in doubt, throw an extra half-hitch may be one philosophy . . . Many surgeons . . . use methods which often seem to be a combination of habit, guesswork and tradition." [37] In essence, knot tying has become almost a static practice within medicine, failing to reflect the availability of new materials, and suffering a lack of evidence-based investigation. The surgeon's knot, square knot and half-hitch are used to the exclusion of all other knots in suture material.

When it comes to ETTs and cloth tape, much is left unsaid. Major textbooks on emergency medicine and procedures mention cloth tape, but not how to tie it [38-41]. The most widely cited knot in journals is the Cow Hitch (Cow), also known as the Lark's Head [15,17,42]. In the authors' experience, this is the knot generally taught to medical students and residents. The main benefit of the Cow Hitch is also its main potential drawback: ease of loosening. Knotting experts consulted for this project suggested it was an unsuitable knot for the task. The Magnus Hitch (Magnus) and Clove Hitch (Clove) were the alternatives the knotting experts suggested, and indeed there is a small body of references in the medical literature also supporting the clove [43-45], although none have subjected it to comparative trials. Rodenberg [13] reported paramedics were securing ETTs using nasal cannula tubing tied with a Clove. If there are differences in performance between the Cow, Clove and Magnus, however, our small sample sizes did not give our study adequate power to detect them.

The authors feel that the major point to draw from the tests using cloth tape is one about materials. Cloth tape outperforms the commercial devices in avoiding breakage (none of the devices survived 10 lb jerks during pilot testing). But slippage and stretch are major weaknesses. The obvious solution is to develop a material that avoids stretch and also provides grip. Such a material would likely provide superior ETT securement. To our knowledge such a material is not yet marketed in a medical context. When a new material becomes available, it may also be worthwhile re-visiting the issue of which knot to tie, and probably in a larger study.

### Limitations

The sample sizes in this study were determined by numbers of units provided by manufacturers, and thus no a priori power analysis was performed. Sample sizes were small. It is quite possible that clinically important differences – for instance, differences in slippage between knots – were found not significant as a result.

This study did not comprehensively include all devices available on the market at the time of the study (December 2002). We invited submission of samples for testing widely. A particularly notable absence was the Hudson™ ETT holder, which along with the Dale^® ^device lead the market. We were supplied with samples of this, but not in time for inclusion in the study.

As previously noted, we did not test adhesive tape in this study.

The protocols used in this study are not the same as those used in FDA testing, or other industrial testing. All the devices in this study have passed testing required by the Food and Drug Administration, conducted with static loads. Loads applied to the ETTs in our study were chosen to provide maximal differentiation in performance. We chose to use dynamic jerks instead of static loads, because we sought to replicate the jerking of an agitated, under-sedated Emergency Department patient. It is possible that this testing exceeds the requirements of real-life practice in the ED, but it is also possible that FDA requirements do not meet the needs of the ED. Most adults can generate a triceps jerk in excess of 10 lbs. There are no data to inform us of whether multiple jerks or single jerks constitute a more valid model for UE.

The jerks applied in this study were uni-directional. This may not accurately replicate the jerking action of an agitated patient.

Commercial devices were not tested at the same loads as cloth tape/knots. This is because the protocols were chosen to provide for maximal differentiation.

Finally, this study did not examine other qualities which are important in any endotracheal tube securement method, such as patient comfort, provision for oral hygiene, and avoidance of trauma to facial and oral tissues.

## Conclusion

In an in vitro model utilizing repeated dynamic jerks of 2.5 lbs and 5 lbs, the Dale^® ^ETT Holder showed less movement than the other devices tested, although not all differences were statistically significant.

There were no statistically significant differences in slippage among the knots in this study, within the constraints of the small sample sizes.

## Competing interests

The authors declare that they have no competing interests. Commercial devices evaluated in this study were donated by manufacturers at the request of the authors. The manufacturers had no involvement in the study.

## Authors' contributions

PL – conception, design, acquisition of data, analysis and interpretation of data, drafting of manuscript, reviewing of manuscript, final approval

AF – conception, design, acquisition of data, reviewing of manuscript, final approval

KS – design, drafting of manuscript, reviewing of manuscript, final approval

PB – analysis and interpretation of data, drafting of manuscript, reviewing of manuscript, final approval

## Pre-publication history

The pre-publication history for this paper can be accessed here:


